# G protein–coupled receptor kinase 6 (GRK6) regulates insulin processing and secretion *via* effects on proinsulin conversion to insulin

**DOI:** 10.1016/j.jbc.2022.102421

**Published:** 2022-08-25

**Authors:** Matthew J. Varney, Wouter Steyaert, Paul J. Coucke, Joris R. Delanghe, David E. Uehling, Babu Joseph, Richard Marcellus, Rima Al-awar, Jeffrey L. Benovic

**Affiliations:** 1 Department of Biochemistry and Molecular Biology, Sidney Kimmel Medical College, Thomas Jefferson University, Philadelphia, Pennsylvania; 2 Department of Human Genetics, Radboud Institute for Molecular Life Sciences, Radboud University Medical Center, Nijmegen, the Netherlands; 3 Center for Medical Genetics, Ghent University and Ghent University Hospital, Ghent, Belgium; 4 Department of Diagnostic Sciences, Ghent University, Ghent, Belgium; 5 Drug Discovery Program, Ontario Institute for Cancer Research, Toronto, Ontario, Canada; 6 Department of Pharmacology and Toxicology, University of Toronto, Toronto, Ontario, Canada

**Keywords:** G protein–coupled receptor, G protein–coupled receptor kinase, insulin, proinsulin, proprotein convertase, type 2 diabetes, BSA, bovine serum albumin, CPE, carboxypeptidase E, DPBS, Dulbecco’s phosphate buffered saline, FBS, fetal bovine serum, GPCR, G protein-coupled receptor, GSIS, glucose stimulated insulin secretion, KRBH, Krebs Ringer bicarbonate buffer with Hepes, MOI, multiplicity of infection, PBS/T, phosphate buffered saline with Tween-20, T2D, type 2 diabetes, TBS/T, Tris buffer saline with Tween-20

## Abstract

Recent studies identified a missense mutation in the gene coding for G protein–coupled receptor kinase 6 (GRK6) that segregates with type 2 diabetes (T2D). To better understand how GRK6 might be involved in T2D, we used pharmacological inhibition and genetic knockdown in the mouse β-cell line, MIN6, to determine whether GRK6 regulates insulin dynamics. We show inhibition of GRK5 and GRK6 increased insulin secretion but reduced insulin processing while *GRK6* knockdown revealed these same processing defects with reduced levels of cellular insulin. *GRK6* knockdown cells also had attenuated insulin secretion but enhanced proinsulin secretion consistent with decreased processing. In support of these findings, we demonstrate GRK6 rescue experiments in knockdown cells restored insulin secretion after glucose treatment. The altered insulin profile appears to be caused by changes in the proprotein convertases, the enzymes responsible for proinsulin to insulin conversion, as *GRK6* knockdown resulted in significantly reduced convertase expression and activity. To identify how the GRK6-P384S mutation found in T2D patients might affect insulin processing, we performed biochemical and cell biological assays to study the properties of the mutant. We found that while GRK6-P384S was more active than WT GRK6, it displayed a cytosolic distribution in cells compared to the normal plasma membrane localization of GRK6. Additionally, GRK6 overexpression in MIN6 cells enhanced proinsulin processing, while GRK6-P384S expression had little effect. Taken together, our data show that GRK6 regulates insulin processing and secretion in a glucose-dependent manner and provide a foundation for understanding the contribution of GRK6 to T2D.

Over 400 million people currently have diabetes, and by 2040, this number is expected to rise to near 700 million (1). Of these cases, approximately 90% are type 2 diabetes (T2D), where developed insulin resistance and increased insulin demand prevents glucose mobilization and provokes pancreatic β-cell dysfunction culminating in insufficient insulin quantities and hyperglycemia (2, 3). The most effective treatment for T2D is lifestyle modifications such as diet and exercise, but for people with genetic predispositions, chronic illness, or poor compliance, this is ineffective and requires pharmacological intervention (1). Current therapies aim to improve insulin resistance (metformin), enhance pancreatic insulin output (meglitinides, sulfonylureas, GLP-1 analogs), or prevent reabsorption of glucose into the blood through the kidneys (SGLT2 inhibitors). These therapies, however, lose their efficacy over time, have dangerous side effects, and utilize discoveries made decades ago regarding β-cell physiology (2, 3, 4, 5). Therefore, it is critical to better understand the molecular mechanisms underlying the etiology of T2D, especially in the pancreatic β-cell, to unveil new avenues for therapeutic intervention to combat the alarming increase in metabolic disease globally.

Insulin processing and secretion in the β-cell is dependent upon a complex array of interconnected regulatory networks that coordinate the proper spatial and temporal release of insulin including insulin granule biogenesis, trafficking, and exocytosis (6). The prototypical signal for insulin production and insulin release is the metabolism of glucose leading to membrane depolarization, calcium influx, and vesicle release (7, 8, 9). However, G protein–coupled receptors (GPCRs) resident on β-cells also play a pivotal role in the regulation of insulin production and secretion (10, 11, 12). GPCRs are a versatile class of cell surface receptors that transmit extracellular stimuli, including peptide hormones and nutrient metabolites, into an intracellular response and are the target of more than 30% of FDA approved drugs (13, 14). Upon agonist binding, these receptors undergo conformational changes that promote heterotrimeric G protein activation initiating various downstream signaling cascades (15). Termination of signaling occurs primarily through the phosphorylation of agonist-activated receptors by GPCR kinases (GRKs). This phosphorylation promotes the recruitment of the adapter protein, β-arrestin, which inhibits G protein coupling and enhances receptor internalization and β-arrestin–mediated signaling (16). Therefore, GRK-mediated phosphorylation of GPCRs functions as a switch to blunt G protein activation and enhance β-arrestin–mediated processes, highlighting their role as important regulators of GPCR function.

There are seven mammalian GRKs classified based on sequence homology and functional features into the GRK1/7, GRK2/3, and GRK4-6 subfamilies. GRK2, 3, 5, and 6 are ubiquitously expressed and serve critical functions in various tissues (17). Specifically in the pancreatic β-cell, these kinases control the extent of GPCR signaling that occurs following activation of the glucagon-like peptide-1 receptor (GLP-1R) and the gastrin inhibitory peptide receptor (GIPR), G_s_-coupled receptors that stimulate insulin secretion (18, 19, 20). In addition, the phosphorylation of agonist occupied M_3_ muscarinic acetylcholine receptors (M_3_AChR) in mouse islets and MIN6 cells recruits β-arrestin2 and enhances second phase insulin secretion (21). Moreover, numerous GPCRs resident on β-cells modulate insulin processing and secretion including the free fatty acid receptors (FFA1-4), glucagon receptor (GCGR), somatostatin receptors (SSTR2 and SSTR5), and several orphan receptors including GPR119 and GPR91 (10, 22, 23, 24). However, surprisingly little is known about how β-cell GRKs regulate G protein– and β-arrestin–dependent mechanisms of insulin secretion through their ability to regulate GPCRs.

In addition to GPCR regulation, emerging evidence suggests potential novel roles for GRKs in islets. For example, Chinese Han T2D patients with a GRK5 polymorphism have an attenuated response to repaglinide, a drug that stimulates insulin secretion by targeting the sulfonylurea receptor (25). It was also reported that β-arrestin1 KO mice exhibit reduced efficacy when treated with tolbutamide and glibenclamide, drugs that also target the sulfonylurea receptor (26). Moreover, MIN6 cells with β-arrestin2 knockdown and mice with β-arrestin2 KO in β-cells have impaired glucose-stimulated insulin secretion (GSIS) from inactivation of calmodulin kinase II (CAMKII) (27). This is similar to that seen in mice with pancreatic deletion of GRK2, where glucose intolerance and diminished insulin secretion is observed (28). Since β-arrestin function is often preceded by GPCR phosphorylation by GRKs, these non-GPCR pathways suggest alternative modes to β-arrestin activation. Together, these findings support the idea that GRK regulation in β-cells may be more complex than just agonist dependent phosphorylation of GPCRs. Thus, identifying mechanisms of GRK action in β-cells should improve our understanding of β-cell pathophysiology contributing to disease.

Recently, Steyaert *et al.* identified a heterozygous missense mutation within the gene coding for GRK6 in two members of a family resulting in a proline to serine change at residue 384 (P384S) (29). The patients presented with hyperproinsulinemia, hyperprogastrinemia, and developed early onset T2D. While genome wide association studies also support a role for GRK6 in T2D (https://t2d.hugeamp.org/. Accessed April 29, 2022), limited data exist defining the mechanism by which GRK6 contributes to β-cell function. To delineate the role of GRK6 in pancreatic β-cells, we performed GSIS assays in MIN6 cells devoid of GRK6 activity. Our results provide important insight on insulin processing that should aid in the advent of new strategies to treat T2D.

## Results

### GRK5/6 inhibition enhances insulin secretion but reduces insulin production in MIN6 cells

**Figure 1:**
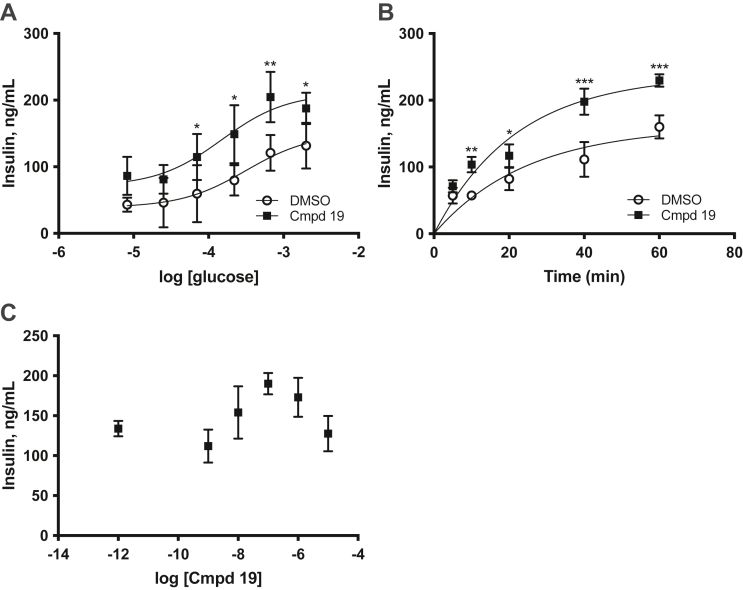
**Treatment with a GRK5/6 inhibitor enhances insulin secretion.***A*, MIN6 cells were incubated in KRBH buffer with low glucose (0.01 mM) for 30 min to equilibrate insulin levels. The cells were washed and KRBH buffer containing 0.01 to 2 mM glucose was added in the absence or presence of 1 μM compound 19 for 1 h. The cell supernatant was then removed and assayed for secreted insulin by ELISA as described in Experimental procedures. *B*, time course of insulin secretion with 1 μM compound 19. *C*, dose response of insulin secretion with compound 19 at 2 mM glucose. The data are represented by the mean ± SD from three independent experiments. Statistical significance was determined using *t* tests. KRBH, Krebs Ringer bicarbonate buffer with Hepes.

Based on the identification of a GRK6 mutation in two patients that have early onset T2D, we wanted to determine whether GRK6 has a role in insulin processing and secretion. To investigate this, we utilized MIN6 cells, a pancreatic β-cell line isolated from a mouse insulinoma that has been used extensively to study β-cell function (30). In these cells, we initially performed GSIS assays in the absence or presence of a 4-aminoquinazoline derivative called compound 19, a GRK inhibitor that was developed as a potential therapeutic to target GRK6 in multiple myeloma (31, 31a). Compound 19 has a low nanomolar potency against GRK5 and GRK6 (IC_50_ values of 5.2 and 2.4 nM, respectively) but does not inhibit GRK2 or GRK3 (IC_50_ > 10 μM) (31, 31a). We found that insulin secretion was augmented about 2-fold after treatment of the cells with 1 μM compound 19 (Fig. 1*A*). We next did a time course of insulin secretion in MIN6 cells in 2 mM glucose with or without compound 19. In cells treated with compound 19, insulin secretion was increased significantly by 10 min compared to control treated cells (Fig. 1*B*). We also performed a dose response with compound 19 and found that increasing concentrations elicited more secretion with 0.1 to 1 μM having the most robust effect (Fig. 1*C*). Thus, these data support a role for GRK5 and/or GRK6 in insulin secretion.

**Figure 2:**
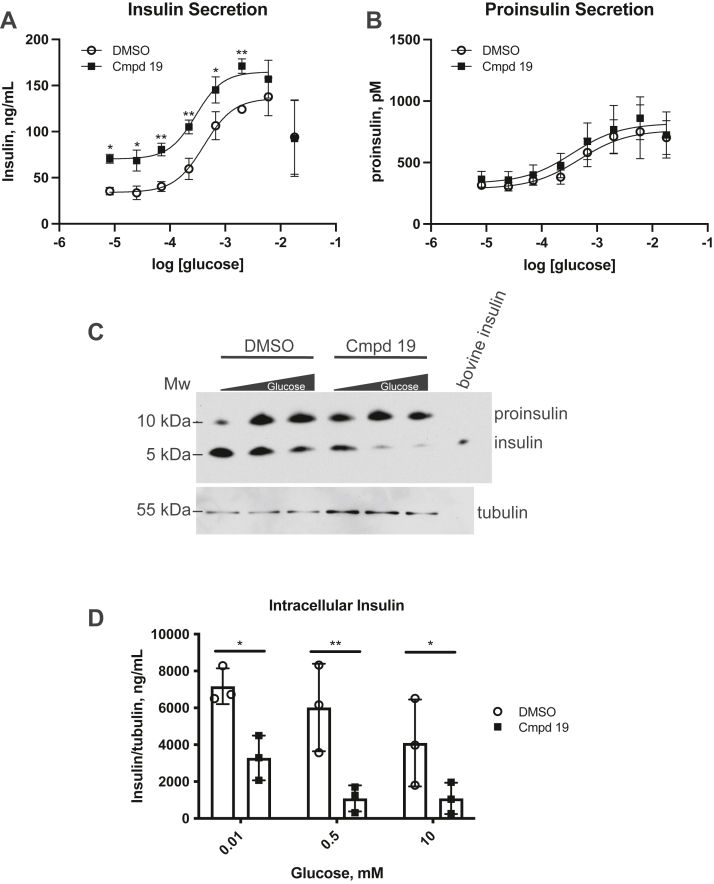
**GRK5/6 inhibition causes a shift in the proinsulin/insulin ratio.** MIN6 cells were incubated in KRBH buffer with low glucose (0.01 mM) for 30 min to equilibrate insulin levels. The cells were then washed and incubated in KRBH buffer containing 0.01 to 18 mM glucose in the presence or absence of 1 μM compound 19 for 1 hour. The amount of secreted insulin (*A*) or proinsulin (*B*) was determined by ELISA as described in Experimental procedures. Statistical significance was determined using *t* tests. *C*, cells incubated for 1 h with glucose (0.01–10 mM) in the presence or absence of 1 μM compound 19 were washed with PBS, lysed, and analyzed by Western blot using an anti-insulin mouse mAb that detects proinsulin and insulin. *D*, the intracellular levels of insulin were determined using ImageJ analysis of Western blots using an anti-insulin rabbit mAb and an insulin protein standard and were normalized to tubulin levels; statistical significance was determined using *t* tests. The data are represented by the mean ± SD from three independent experiments. Tubulin was used as a protein loading control. KRBH, Krebs Ringer bicarbonate buffer with Hepes.

To compare insulin and proinsulin secretion, we performed GSIS assays at higher glucose concentrations since proinsulin secretion is generally shifted to the right in its glucose dose response compared to insulin (32). We again observed the increase in insulin secretion with compound 19 treatment, although this effect was lost at 18 mM glucose (Fig. 2*A*). This is not surprising since high glucose concentrations can lead to impaired insulin secretion (33, 34). While we expected to see a comparable increase in proinsulin secretion since the relative insulin to proinsulin composition from granule to granule is maintained (35), we did not observe a significant increase after compound 19 treatment (Fig. 2*B*). This suggests that the internal components comprising the secretory granule were being compromised since the relative level of proinsulin being secreted did not match that of insulin. We investigated this by examining the proinsulin/insulin (P/I) ratio by Western blot using antibodies that can detect both proinsulin (10 kDa) and insulin (6 kDa). This enabled us to examine the intracellular P/I ratio in MIN6 cells with or without compound 19 treatment. Interestingly, we identified a striking difference in the P/I ratio in MIN6 cells that shifted strongly in favor of proinsulin in the presence of compound 19 (Fig. 2*C*). Additionally, the cellular level of insulin was reduced dramatically in inhibitor-treated cells compared to control cells (Fig. 2*D*). Taken together, these data suggest that GRK5 and/or GRK6 enhance insulin synthesis but have a negative impact on insulin secretion. This provides an interesting conundrum to better understand the role of these kinases on insulin dynamics.

### Knockdown of GRK6 in MIN6 cells diminishes insulin production and secretion

**Figure 3:**
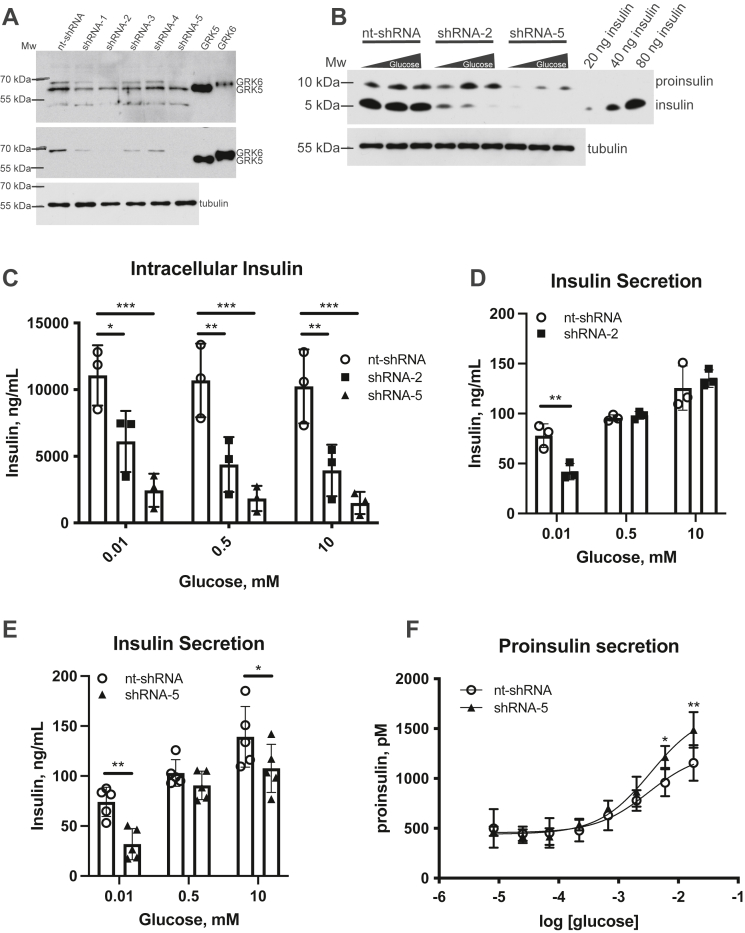
**GRK6 knockdown attenuates insulin processing and increases proinsulin secretion.***A*, MIN6 cells were infected with five different GRK6 shRNA lentiviral constructs targeting different regions of the gene for 48 h and clones established as described in Experimental procedures. The cells were then lysed and assayed by Western blot using GRK4-6 (top image) and GRK6 specific (middle image) antibodies to determine the extent of GRK6 knockdown. Nt-shRNA = nontargeting-shRNA. *B*, the nt-shRNA, shRNA-2, and shRNA-5 MIN6 cell lines were incubated in KRBH buffer with low glucose (0.01 mM) for 30 min to equilibrate insulin levels. Cells were washed, incubated with KRBH buffer containing 0.01 to 10 mM glucose for 1 h, and then washed with PBS, lysed, and assayed by Western blot using an anti-insulin mouse mAb that detects proinsulin and insulin. *C*, the levels of cellular insulin were determined using ImageJ analysis of Western blots using an anti-insulin rabbit mAb and an insulin protein standard. Statistical analysis was determined using two-way ANOVA with a post hoc Tukey’s multiple comparisons test. The amount of insulin secreted in shRNA-2 (*D*) or shRNA-5 (*E*) knockdown cells was determined by ELISA as described in Experimental procedures. Statistical significance was determined using *t* tests. *F*, proinsulin secretion in shRNA-5 knockdown cells was determined by ELISA as described in Experimental procedures. Statistical significance was determined using *t* tests. The data are represented by the mean ± SD from three to five independent experiments. Tubulin was used as a protein loading control. KRBH, Krebs Ringer bicarbonate buffer with Hepes.

While compound 19 treatment increased insulin secretion and impaired insulin synthesis, we do not know which of these changes are due to GRK6 inhibition, if any, since the compound also inhibits GRK5 (31, 31a). To assess the specific role of GRK6, we used shRNA knockdown of GRK6 in MIN6 cells. We utilized five different lentiviral shRNA sequences, each targeting a different region of the GRK6 gene to generate stable cell lines. All five lines had reduced GRK6 expression (from ∼50% to 90%) compared to control shRNA treated cells while there was no appreciable effect on GRK5 expression (Fig. 3*A*).

We selected the two cell lines with the highest GRK6 suppression, shRNA-2 and shRNA-5, and treated them with different glucose concentrations to determine the role of GRK6 on insulin dynamics. There was a prominent shift in the P/I ratio favoring proinsulin in the two knockdown cell lines compared to control cells (Fig. 3*B*). Additionally, the cellular levels of insulin were severely attenuated at all glucose concentrations in both shRNA-2 and shRNA-5 cells compared to control cells (Fig. 3*C*). Specifically, insulin levels at 10 mM glucose were decreased by 62% and 85% in shRNA-2 and shRNA-5 cells, respectively. Next, we investigated how insulin secretion was affected by GRK6 knockdown. In shRNA-2 cells, insulin secretion was attenuated only at the lowest glucose concentration (Fig. 3*D*) while shRNA-5 cells had a significant reduction at the lowest and highest glucose concentrations (Fig. 3*E*). Furthermore, proinsulin secretion was significantly increased in shRNA-5 cells at higher glucose concentrations compared to control cells (Fig. 3*F*). The reduced insulin secretion and increased proinsulin secretion we see in these knockdown cells mirrors the intracellular changes we see in the proteins. These data support a role for GRK6 in insulin synthesis that ultimately contributes to secretion. Furthermore, the increased insulin secretion seen with compound 19 treatment (Fig. 1) might be due to inhibition of GRK5 or possibly other targets since this is not observed in the MIN6 cells with specific GRK6 knockdown.

### Effect of GRK6 knockdown on expression and activity of the prohormone converting enzymes PC1, PC2, and CPE in MIN6 cells

Metabolic hormones, including insulin, GLP-1, and glucagon, are made from the sequential cleavage of their respective prohormones by different combinations of the prohormone-converting enzymes and carboxypeptidase E (CPE) (36). Insulin is produced in secretory vesicles by sequential cleavage of proinsulin by prohormone convertase 1 (PC1), prohormone convertase 2 (PC2), and CPE (6, 37). These enzymes exist as proproteins and self-activate to form mature converting enzymes capable of producing insulin (38). The activity of these enzymes is dependent upon glucose and other signaling pathways to create an environment within the vesicle suitable for enzymatic activity (39, 40). These include changes in vesicle pH and calcium as well as transregulation by small peptides (41, 42). Thus, the amount and activity of the converting enzymes is regulated by signaling pathways elicited by glucose and other stimuli like GLP-1 (43, 44).

**Figure 4:**
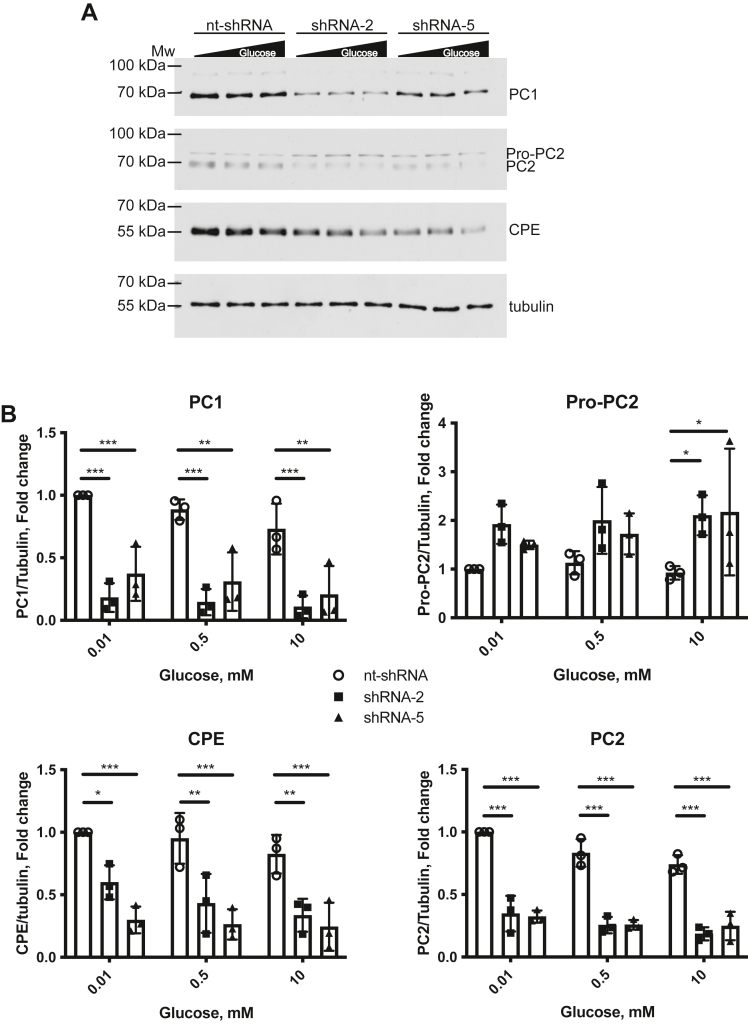
**GRK6 knockdown in MIN6 cells reduces the levels of the prohormone convertases PC1, PC2, and CPE.***A*, MIN6 cells with GRK6 knockdown were incubated in KRBH buffer with low glucose (0.01 mM) for 30 min to equilibrate insulin levels. The cells were washed, KRBH buffer containing 0.01 to 10 mM glucose was added for 1 h, and the cells were washed with PBS, lysed, and analyzed by Western blot using antibodies against PC1, PC2, CPE, and tubulin. *B*, the relative cellular levels of PC1, PC2, and CPE were normalized to the control treatment and tubulin levels using ImageJ analysis. The data are represented by the mean ± SD from three independent experiments. Statistical significance was determined using two-way ANOVA with a post hoc Tukey’s multiple comparisons test. Tubulin was used as a protein loading control. CPE, carboxypeptidase E; KRBH, Krebs Ringer bicarbonate buffer with Hepes.

Since we are seeing a clear diminution in the conversion of proinsulin to insulin, we hypothesized that reducing GRK6 levels is inhibiting the ability of the converting enzymes to produce mature insulin. To test this, we treated knockdown cells with glucose for 1 hour and then determined the protein level of PC1, PC2, and CPE. We saw a significant reduction in PC1, PC2, and CPE in both shRNA-2 and shRNA-5 cells compared to control shRNA cells (Fig. 4, *A* and *B*). We also saw a striking shift in the ratio of inactive to active PC2 that was most prevalent at 10 mM glucose (Fig. 4*B*). This suggests that the environment in the GRK6 knockdown cells is not optimal for PC2 activation. Thus, GRK6 knockdown attenuates expression of all three converting enzymes consistent with a processing defect contributing to reduced insulin synthesis.

### GRK6 re-expression in knockdown MIN6 cells rescues insulin secretion

**Figure 5:**
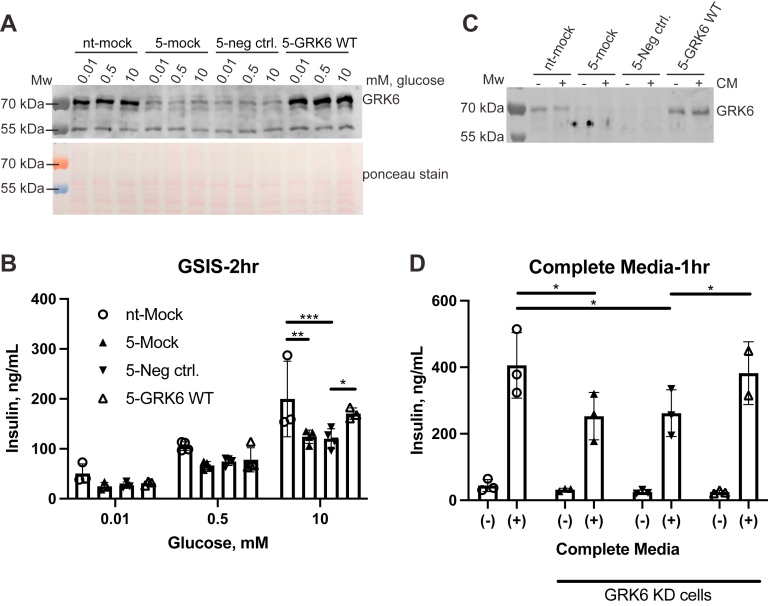
**GRK6 rescue in knockdown cells restores insulin secretion after high glucose treatment.***A*, GRK6 knockdown cells were infected with lentivirus encoding human GRK6 at 2 MOI for 24 h. Each infected well was resuspended and transferred to three new wells and allowed to grow for an additional 48 h. The cells were put in low glucose (0.01 mM) KRBH buffer to equilibrate insulin levels for 30 min, washed, and KRBH buffer containing 0.01 to 10 mM glucose was added for 2 h. The level of GRK6 expression was determined by lysing the cells and Western blotting using an anti-GRK6 antibody. *B*, insulin secretion was measured by ELISA as described in Experimental procedures prior to lysis. Statistical significance was determined using two-way ANOVA with a post hoc Tukey’s multiple comparisons test. The data are represented by the mean ± SD from three to four independent experiments. *C*, GRK6 knockdown cells were infected with 1.7 MOI lentivirus encoding human GRK6 for 48 h. Each infected well was resuspended and transferred to two new wells and allowed to grow for an additional 24 h. The level of GRK6 expression was evaluated by Western blotting. *D*, cells were equilibrated in low glucose (0.01 mM) KRBH buffer for 30 min and then washed and treated with either low glucose buffer (−) or complete medium (+) for 1 h. Cell media was removed and assayed by ELISA to determine the level of secreted insulin. Statistical significance was determined by two-way ANOVA. The data are represented by the mean ± SD from three independent experiments. Ponceau staining was used as a protein loading control. KRBH, Krebs Ringer bicarbonate buffer with Hepes; MOI, multiplicity of infection.

To confirm that the effects of GRK6 knockdown on insulin secretion are specific to GRK6 function, we performed rescue experiments to reintroduce GRK6 into our knockdown cells. We used a lentivirus-based system to increase expression efficiency and be able to titrate GRK6 expression to be near endogenous levels (Fig. 5*A*). We then performed GSIS experiments in shRNA-5 cells with or without GRK6 re-expression. We monitored insulin secretion for our rescue experiments because restoration of insulin secretion indicates that the cell is effectively producing and processing insulin that can then be released. Since we did not see any rescue of secretion after a 1 h treatment with glucose, we postulated that a longer glucose treatment might provide better recovery as it may take time for enough proinsulin to be processed to allow for more insulin synthesis and subsequent secretion. In a glucose-only treatment, the secondary amplified phase of insulin secretion is initiated and maintained by various metabolite signaling pathways that occur as early as 10 min after stimulation and can last for hours (7, 45, 46). Therefore, recovery of insulin secretion by GRK6 may be time dependent and take more than an hour for the relevant pathway to become a factor contributing to secretion. In addition, although we have good GRK6 expression, the entire population of MIN6 cells is likely not expressing GRK6, so only the population of GRK6-expressing cells will contribute to the rescue. After 2 h of glucose treatment, we saw reduced insulin secretion in both noninfected and negative control lentivirus-infected shRNA-5 cells compared to the control shRNA cells as expected. While GRK6 lentivirus-infected cells did not rescue insulin levels at 0.01 mM and 0.5 mM glucose, GRK6 re-expression was able to restore insulin secretion to near normal levels at 10 mM glucose (Fig. 5*B*). Thus, these data support our knockdown studies that GRK6 regulates insulin secretion and does so in a glucose and time-dependent manner.

We also performed rescue experiments using complete medium treatment for 1 h to elicit robust stimulation of insulin secretion. This was done in shRNA-5 cells that had GRK6 re-expression at near endogenous levels (Fig. 5*C*). GRK6 knockdown cells had reduced insulin secretion after complete medium treatment that was then restored with GRK6 expression (Fig. 5*D*). This further reinforces the positive role GRK6 has on insulin secretion and suggests other amplifying signals in addition to glucose that help to accelerate insulin production and release after GRK6 re-expression.

### Activity and localization of GRK6-P384S

**Figure 6:**
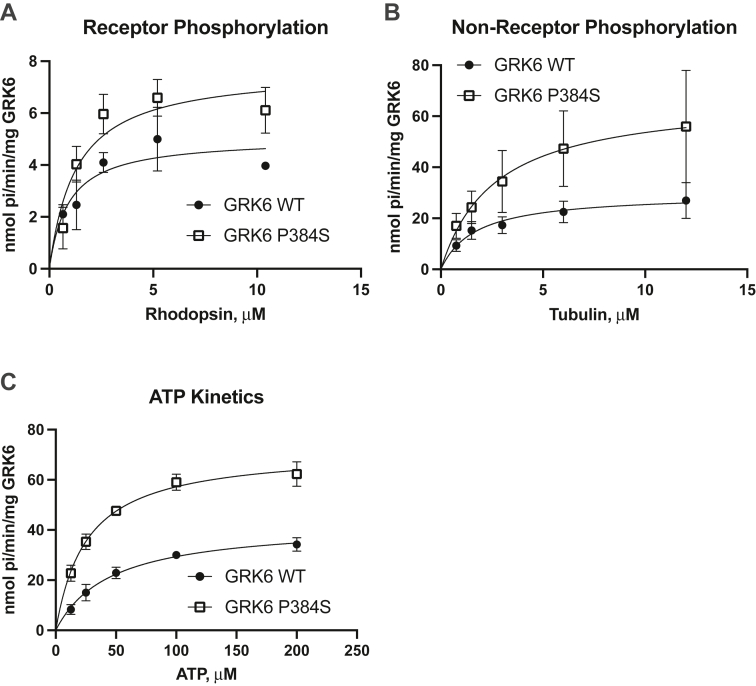
***In vitro* phosphorylation of receptor and nonreceptor substrates by WT GRK6 and GRK6-P384S *in vitro*.***A*, Sf9 cell expressed and purified WT GRK6 and GRK6-P384S were used to phosphorylate varying concentrations of light-activated rhodopsin for 5 min at 30 °C using 30 nM kinase and radiolabeled ATP as described in Experimental procedures. Radioactivity was determined by scintillation counting of protein bands excised from an SDS-PAGE gel. *B*, tubulin phosphorylation determined as described previously using tubulin as substrate. *C*, ATP kinetics were determined as described previously but using a reaction time of 3 min and tubulin as the substrate. The data are represented by the mean ± SD from three (*A* and *B*) or four (*C*) independent experiments for each substrate tested and were subjected to Michaelis–Menten kinetic analysis.

We next wanted to better understand how the GRK6-P384S mutation that was found in the patients with T2D affects GRK6 function. Proline 384 lies within the large lobe of the catalytic domain close to the substrate-binding site (47). To evaluate activity, we expressed human GRK6 and GRK6-P384S in Sf9 insect cells, purified the proteins, and then studied their ability to phosphorylate receptor and nonreceptor substrates. GRK6-P384S had increased catalytic activity for all substrates tested with a higher *V*_max_ for rhodopsin (Fig. 6*A*), tubulin (Fig. 6*B*), and ATP (Fig. 6*C*). Concomitantly, the catalytic efficiency (*k*_cat_/*K*_*m*_) of the mutant enzyme was increased in all cases with a 3-fold increase with ATP (Table 1). Taken together, these studies show that the mutation increases the catalytic activity of GRK6 regardless of the substrate.

**Table 1:** GRK6 WT and GRK6 P384S Michaelis–Menten kinetic analysis

Kinetic constants	Rhodopsin		Tubulin		ATP	
	GRK6 WT	GRK6 P384S	GRK6 WT	GRK6 P384S	GRK6 WT	GRK6 P384S
*V*_max_, nmol Pi/min/mg	5.0 ± 0.5	7.7 ± 0.7	29.7 ± 3.1	67.7 ± 12.0	42.7 ± 2.0	72.0 ± 2.1
*K*_*m*_, μM	0.92 ± 0.38	1.3 ± 0.4	1.7 ± 0.6	2.6 ± 1.3	45.5 ± 5.9	25.8 ± 2.5
*k*_cat_, s^−1^	5.6 × 10^−3^ ± 0.6 × 10^−3^	8.6 × 10^−3^ ± 0.8 × 10^−3^	33.0 × 10^−3^ ± 3.5 × 10^−3^	75.3 × 10^−3^ ± 13.4 × 10^−3^	47.5 × 10^−3^ ± 2.3 × 10^−3^	80.0 × 10^−3^ ± 2.4 × 10^−3^

The data are represented by the mean ± SEM from three independent experiments for each substrate and were subjected to Michaelis–Menten kinetic analysis.

**Figure 7:**
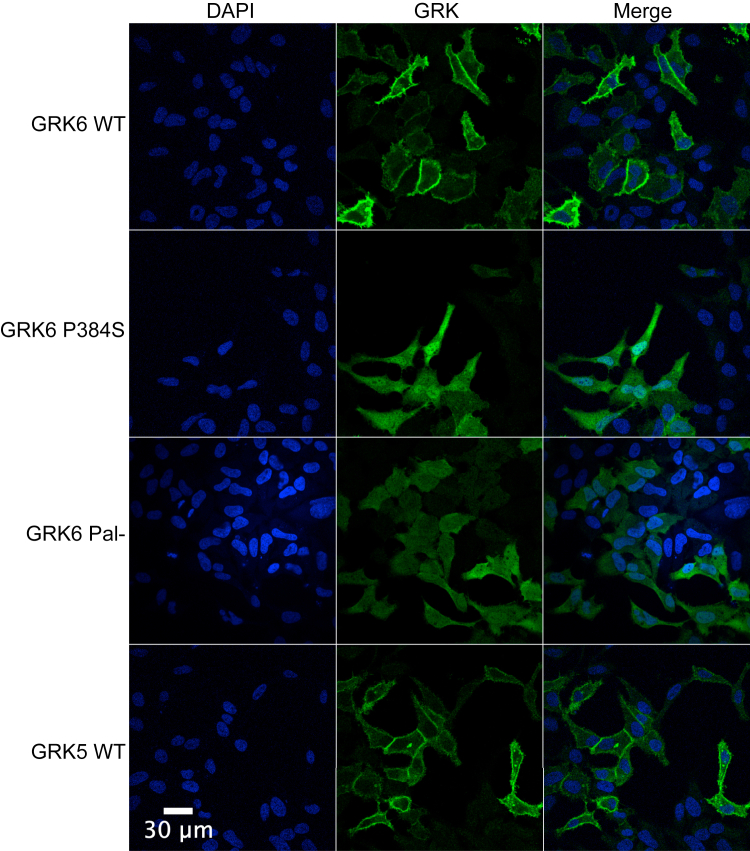
**GRK localization in HeLa cells.** Cells were transiently transfected with the indicated pcDNA3 constructs for 48 h. The cells were then fixed, permeabilized, blocked, and stained with mouse anti-GRK4-6 primary antibody (Millipore) and Alexa-Fluor 488–conjugated antimouse secondary antibody. Images were taken using a Nikon A1R fluorescent microscope in the Sidney Kimmel Cancer Center bioimaging facility. Images are representative of three independent experiments. GRK6 Pal- is a palmitoylation deficient GRK6 with cysteine residues 561, 562, and 565 mutated to serine.

In addition to the P384S mutation being near the catalytic active site, it is also three residues N-terminal to the nuclear localization signal in GRK6 (47). Therefore, we determined if the localization of the mutant kinase was perturbed. Both GRK5 and GRK6 are normally at the plasma membrane, GRK5 *via* polybasic regions and GRK6 *via* C-terminal palmitoylation (48, 49, 50). As expected, we observed GRK5 and GRK6 almost exclusively at the plasma membrane when expressed in HeLa cells (Fig. 7). GRK6 Pal-, which has three C-terminal cysteine residues mutated to alanine to inhibit palmitoylation, was cytosolically distributed as previously reported (51). Surprisingly, GRK6-P384S was primarily in the cytoplasm as well as in the nucleus (Fig. 7). This localization was distinct from GRK6 Pal-, which was not as prevalent in the nucleus. This suggests an additional mechanism to palmitoylation controlling GRK6 dynamics between the plasma membrane and other subcellular regions (51).

**Figure 8:**
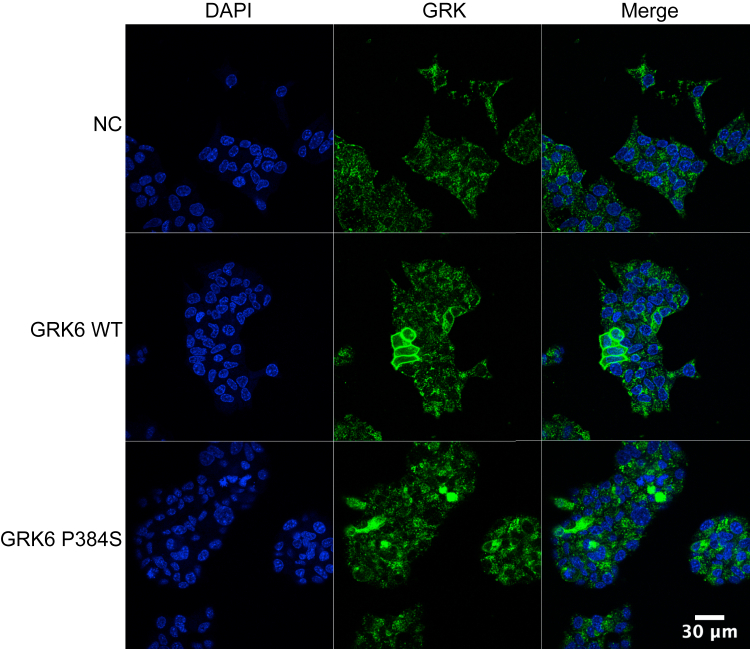
**GRK6 localization in MIN6 cells.** nt-shRNA cells were infected with the indicated lentiviruses at 2 MOI. After 24 h, the cells were trypsinized and moved to new plates with sterile coverslips for an additional 48 h. The cells were then fixed, permeabilized, blocked, and stained with rabbit anti-GRK6 primary antibody and Alexa-Fluor 488–conjugated anti-rabbit secondary antibody. Images were taken using a Nikon A1R fluorescent microscope in the Sidney Kimmel Cancer Center bioimaging facility. NC is the negative control virus.

While HeLa cells are often used for immunofluorescence studies due to their favorable imaging properties, we also investigated the expression of GRK6 in MIN6 cells. Using our control knockdown MIN6 cells, we saw a punctate staining pattern when using a GRK6-specific antibody (Fig. 8). We believe this is primarily nonspecific staining since we see a similar punctate pattern in our shRNA-2 and shRNA-5 GRK6 knockdown cells (not shown). To evaluate the localization of GRK6 and GRK6-P384S in MIN6 cells, we infected the cells with WT or mutant GRK6 lentiviruses, and the cells were then imaged after 72 h. GRK6 exhibited strong plasma membrane localization while GRK6-P384S was primarily cytosolic, consistent with its localization in HeLa cells (Fig. 8). It was also evident that only 25% to 30% of the cells significantly overexpressed GRK6 or GRK6-P384S. This low efficiency of expression might help to explain the small but statistically significant rescue in insulin secretion with GRK6 re-expression (Fig. 5, *B* and *D*). Taken together, the GRK6-P384S mutant alters the localization of the kinase in both HeLa and MIN6 cells, which might contribute to the phenotype observed in the T2D patients with the mutation.

### GRK6 overexpression in MIN6 cells increases intracellular insulin levels

We next studied whether GRK6 directly affects insulin processing and production in MIN6 cells following glucose treatment. We know that inhibition and knockdown of GRK6 has a profound effect on insulin synthesis, but we did not see effective rescue of intracellular insulin levels after a 2 h glucose treatment as we did with insulin secretion. Newly made insulin is preferentially released as opposed to stored insulin, which likely was a contributing factor in the rescue of insulin secretion since secretion was restored without a parallel increase in intracellular insulin (52). Therefore, we proposed that treatments longer than 2 h might produce a more profound effect since the second amplified phase of insulin secretion is dependent upon various metabolic-coupling pathways that act to support insulin secretion and insulin production and that 2 h is not enough time to observe increased insulin production (7, 45, 46). We used WT MIN6 cells with endogenous GRK6 for these experiments for two reasons. The first is to try to recapitulate the genotype of the patients who have both WT GRK6 and GRK6-P384S expression to best study the effect this mutation is having in these individuals. The second is to try to determine which properties of GRK6, including catalytic activity and membrane localization, are critical for regulating insulin production. Therefore, we used two mutants in addition to WT GRK6, GRK6-P384S and catalytically inactive GRK6-K215R, to help determine if membrane localization and/or catalytic activity contribute to normal insulin processing (53).

**Figure 9:**
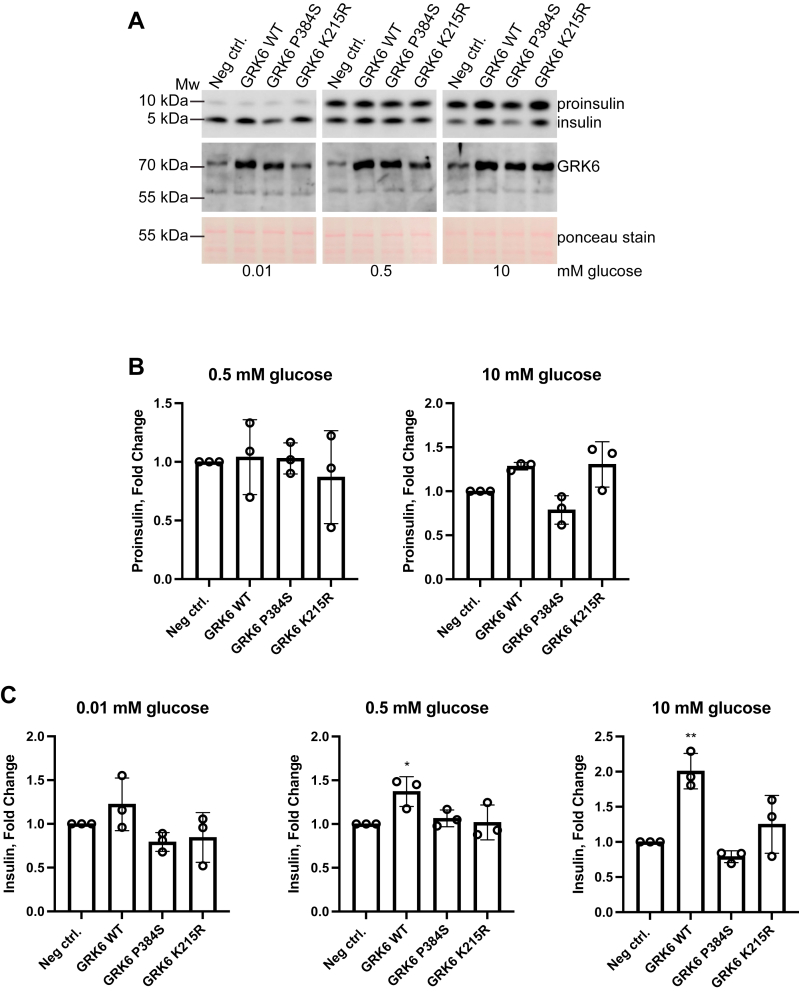
**Overexpression of WT GRK6 but not GRK6-P384S augments glucose-promoted insulin production.***A*, MIN6 cells were infected with either negative control lentivirus or lentivirus encoding human GRK6 at 2 MOI for 24 h. Each infected well was resuspended and transferred to three new wells and allowed to grow for an additional 48 h. MIN6 cells were then put in low glucose (0.01 mM) KRBH buffer to equilibrate insulin levels for 30 min, cells were washed, and KRBH buffer containing 0.01 to 10 mM glucose was added for 4 h. The cells were then lysed and the levels of insulin, proinsulin, and GRK6 were determined by Western blotting. The levels of proinsulin (*B*) and insulin (*C*) relative to the negative control treatment were determined using the Amersham Bioimager quantitation software to measure the relative intensities of each protein band. Statistical significance was determined by one-way ANOVA with a post hoc Dunnett’s multiple comparisons test. The analysis was done using an anti-insulin mouse mAb. The data are represented by the mean ± SD from three independent experiments. Ponceau staining was used as a protein loading control. KRBH, Krebs Ringer bicarbonate buffer with Hepes; MOI, multiplicity of infection.

We expressed GRK6, GRK6-P384S, and GRK6-K215R in MIN6 cells using specific lentiviruses and then treated the cells for 4 h with varying concentrations of glucose (Fig. 9*A*). Proinsulin biosynthesis was not affected at 0.5 mM glucose but increased at 10 mM glucose for both GRK6 and GRK6-K215R (Fig. 9*B*). In contrast, proinsulin levels in cells expressing GRK6-P384S were unresponsive to glucose treatment. There was a large increase in the amount of intracellular insulin when GRK6 was overexpressed in the presence of 10 mM glucose while there were smaller increases at 0.5 mM and 0.01 mM glucose, indicating a glucose-dependent increase controlled by GRK6 (Fig. 9*C*). Strikingly, GRK6-P384S exhibited no augmentation in insulin processing at any glucose concentration and, in fact, modestly reduced insulin levels at 0.01 and 10 mM glucose. Similarly, the kinase dead mutant was also largely unresponsive to glucose (Fig. 9*C*). Therefore, WT GRK6 had the best improvement in the P/I ratio and was more sensitive to glucose than the mutants. Together, these data show that GRK6 can augment intracellular insulin levels in a glucose-dependent manner and that membrane localization and catalytic activity are important for this function. Additionally, it suggests that a combination of WT GRK6 and GRK6-P384S expressed in the pancreatic β-cell can inhibit normal insulin dynamics since proinsulin biosynthesis is being obstructed in conjunction with its processing to insulin. Taken together, our findings indicate that GRK6 is necessary for optimal insulin production and secretion after glucose stimulation.

## Discussion

In the present study, we provide evidence that GRK6 is a positive regulator of insulin processing and secretion in β-cells. Both inhibition and shRNA-mediated knockdown of GRK6 shifted the P/I ratio toward proinsulin and reduced intracellular insulin levels. In GRK6 knockdown cells, these effects were accompanied by reduced insulin secretion but enhanced proinsulin secretion reflecting the intracellular changes seen in the hormone. Consistent with the insulin processing defect, there was a reduction in the activity and expression of the prohormone-converting enzymes necessary for proinsulin to insulin conversion. To confirm our knockdown studies, GRK6 rescue was employed where GRK6 re-expression was able to restore insulin secretion after 2 h of GSIS. We also found that GRK6-P384S is distributed in the cytosol as opposed to the plasma membrane and that it is incapable of augmenting insulin production in MIN6 cells after prolonged glucose treatment. Taken together, our data show that GRK6 regulates insulin processing and secretion through its ability to support proinsulin biosynthesis and the function of the prohormone convertases to effectively convert proinsulin to insulin.

GRK2 and GRK5 have already been proposed as potential drug targets in metabolic disease including T2D (25, 54). However, this has not been explored in detail for GRK6 where only genetic associations with the disease exist. Steyaert *et al*. recently reported a mutation in GRK6 identified in two patients who developed early onset T2D (29). This mutation, P384S, lies within the large lobe of the catalytic domain of GRK6, suggesting that it may impact GRK6 function (47). Using whole exome sequencing, the authors determined that this mutation cosegregated with the disease (29). The phenotype observed in the GRK6-P384S patients suggested that they have a compromised ability to process and secrete mature insulin. The inability of these patients to process prohormones is also supported by the concomitant increase in progastrin originating from G-cells of the stomach and pro-FGF produced in osteocytes found in bone (29, 47, 55, 56).

Genetic evidence from the Type 2 Diabetes Knowledge Portal has identified single nucleotide polymorphisms in GRK6 associated with T2D and other related metabolic parameters. This online database provides information on the strength of an association between a gene and a disease or trait based on the available human genetic datasets referred to as the Human Genetic Evidence Calculator (HuGE). Any score above 1 indicates an association with a disease with higher numbers having a stronger association (57). When comparing the link of GRKs to fasting glucose levels, GRK5 and GRK6 have HuGE scores of 3.33 and 1.12, respectively, whereas GRK2 is less than 1. Although there is not yet genome wide significant associations of GRK6 in T2D, the genetic evidence presented here for GRK6 in combination with other GRKs as viable drug targets provided the foundation for functional investigation of GRK6 in pancreatic β-cells.

We report that GRK6 facilitates insulin secretion in a glucose- and time-dependent manner. It is clear from these studies that ablating GRK6 function either pharmacologically or genetically abrogates insulin production and alters the P/I ratio to favor proinsulin. Additionally, the activity and expression of the converting enzymes that is lost is more pronounced at higher glucose concentrations. Combined with the fact that secreted insulin but not intracellular insulin was restored with GRK6 rescue after 2 h of glucose treatment, these data indicate that GRK6 is assisting new insulin synthesis possibly by enhancing translational mechanisms of preexisting mRNA pools of secretory granule proteins or by creating a secretory vesicle environment suitable for proinsulin to insulin conversion. This is supported by the increase in intracellular proinsulin and insulin seen after GRK6 overexpression in MIN6 cells after 4 h of glucose stimulation, which would not be enough time to elicit meaningful changes in preproinsulin mRNA levels that might contribute to new insulin translation. This requires approximately 24 h of chronic glucose stimulation before the cell upregulates transcription of preproinsulin mRNA to replenish its mRNA reserve since the β-cell already keeps it in excess for acute stimulations (58). Consequently, our data suggest that GRK6 is having a more prominent role in regulating the activity of the converting enzymes. This is because we see an accumulation of inactive PC2 as well as lower levels of active PC1, PC2, and CPE in GRK6 knockdown cells. In addition, the P/I ratio under conditions where GRK6 function is reduced are always shifted toward proinsulin regardless of the absolute amounts of proinsulin and insulin. Therefore, less of the available proinsulin is being converted to insulin in MIN6 cells with attenuated GRK6 function. The exact opposite is true in MIN6 cells overexpressing WT GRK6 where P/I ratios are shifted toward insulin, further supporting GRK6-mediated control of prohormone convertase function that ultimately controls insulin production.

Using GRK6 mutants, we found that membrane localization is a critical determinant to enhancing insulin production in MIN6 cells since GRK6-P384S overexpression had no effect. Overexpression of WT GRK6 elevated insulin synthesis ∼2-fold, whereas GRK6-K215R was largely unresponsive to glucose. From these data, we conclude that the ability of GRK6 to increase insulin production is dictated by its ability to be at the plasma membrane and phosphorylate a substrate, presumably a GPCR. In GRK6 knockdown cells, such phosphorylation would be lost and could lead to reduced insulin processing and secretion through increased G-protein or reduced β-arrestin signaling. With less receptor phosphorylation, G_i_ proteins could become overactive and prevent cAMP accumulation and insulin secretion by attenuating the secretory vesicle environment suitable for proinsulin to insulin conversion. Conversely, this lack of receptor phosphorylation could also inhibit β-arrestin recruitment and β-arrestin–mediated signaling pathways that support insulin secretion as has already been described for receptors including the M_3_AChR, GLP-1R, and FFA1 (21, 59, 60).

We have not identified potential substrates for GRK6 that act to regulate insulin processing and secretion. It is known, however, that intracellular cAMP and calcium signaling regulate insulin production and its secretion, so it is tempting to speculate that GPCR signaling activated through the metabolism of glucose may be an additional mechanism by which the β-cell controls its insulin secretion and that GRK6 is able to modulate these pathways (46). The amplified phase of insulin secretion, otherwise known as the K_ATP_ channel-independent pathway, is a sustained long-term release of insulin that can last for hours and accounts for the majority of insulin secreted over long periods (45). The regulatory mechanisms supporting the amplified phase of insulin secretion are not fully defined but involve glycolytic and anaplerotic byproducts of glucose metabolism that are endogenous agonists for numerous β-cell GPCRs. For example, acetate, a waste product of glycolysis and gut microbiota metabolism, stimulates FFA2 and FFA3, which are G_i_ coupled (FFA2 also couples to G_q_) and inhibit insulin secretion. Succinate, a citric acid cycle intermediate, stimulates the SUCCR, thus modulating proinsulin biosynthesis and insulin secretion (9, 10, 23). Glucose stimulation can also initiate β-cell lipolysis where the resulting long chain FFAs can activate β-cell FFA1 subsequently activating PKC and PKD, kinases implicated in insulin secretion (61, 62). FFA1 and FFA4 agonism have become primary targets for the pharmaceutical industry due to the ability of these receptors to augment insulin secretion in cell and animal models that have been limited by toxic side effects (63, 64). Therefore, future studies identifying GRK6 substrates in the β-cell should illuminate the GRK6-dependent pathways contributing to insulin processing and secretion and whether phosphorylation is affecting G-protein– or β-arrestin–mediated signaling.

In conclusion, we have found an important role for GRK6 in controlling insulin production and secretion in a time- and glucose-dependent manner in MIN6 cells. For decades, the same approaches to treat T2D have been utilized largely revolving around various GLP-1R agonists and improving insulin resistance (4, 5). A mutation in the gene coding for GRK6 prompted us to explore novel mechanisms regulated by GRKs in the β-cell revealing a new role for GRK6 in insulin processing and secretion. These data help to support the notion that β-cell biology is extremely complex and that a thorough understanding of GPCR signaling and its regulatory components including the GRKs can lead to previously unappreciated and druggable cellular systems to better treat T2D.

### Limitations of study

Our work provides important insight into the regulation of insulin processing and secretion mediated by GRK6 in the pancreatic β-cell. Pancreatic β-cells are a complex metabolic hub that respond to numerous hormones and nutrient-derived metabolites in addition to glucose that both stimulate and inhibit insulin production and secretion. Using isolated pancreatic β-cell lines such as MIN6 cells is a basic approach to initially investigate a protein or signaling pathway to understand its role in insulin dynamics and is used extensively in the field of diabetes research. MIN6 cells and other isolated rodent cell lines are used because isolated human β-cells did not exist until recently and are not that well characterized like the decades of research using various rodent β-cell lines and their likeness to human β-cell physiology. Nonetheless, β-cells exist in islets with α-cells and δ-cells, and these islets exist in whole animals within the pancreas that are subject to further complexities characteristic of living systems. Therefore, studies using isolated human/mouse islets or animal models with GRK6 KO would be useful tools to further understand the physiological role of GRK6 in T2D and its relevance to humans. Furthermore, testing whether GRK6-P384S heterozygous knock-in mice recapitulate the patient phenotype would help to better understand the mechanistic role of GRK6-P384S in T2D and its contribution in the patients with this mutation. These approaches would help to extend our data in isolated β-cells.

## Experimental procedures

### Generation of GRK6-P384S

A GRK6-P384S expression construct was created by site-directed mutagenesis of human GRK6 using the Quikchange II site-directed mutagenesis kit (Agilent Technologies) using antisense (5′-cctctgctggaagctcgactggcctgcg-3′) and sense (5′-cgcaggccagtcgagcttccagcagagg-3′) primers from Integrated DNA Technologies. The mutation was confirmed by DNA sequencing.

### Cell culture

MIN6 cells were maintained in Dulbecco’s modified Eagle’s medium (DMEM, GIBCO) lacking L-glutamine and supplemented with 15% fetal bovine serum (FBS, Corning), penicillin–streptomycin–glutamine (GIBCO), and 55 μM 2-mercaptoethanol (GIBCO). MIN6 cells with stable shRNA knockdown were maintained in DMEM (GIBCO) lacking L-glutamine supplemented with 15% FBS, L-glutamine (Corning), 55 μM 2-mercaptoethanol, and 2.5 μg/ml puromycin (GIBCO). Cells were passaged every 2 to 3 days and all experiments used cells in P25-P38. HeLa cells were maintained in DMEM (Corning) supplemented with 10% FBS and penicillin–streptomycin (Corning) and passaged every 2 to 3 days. Cells were checked periodically for mycoplasma contamination using the Lookout Mycoplasma PCR Detection Kit (Sigma). All cell lines were maintained at 37 °C and 5% CO_2_ in a humidified incubator.

### Expression and purification of GRK6

The Bac-to-Bac Baculovirus Expression System (Invitrogen) was used to generate baculoviruses to express human GRK6 and GRK6-P384S in Sf9 cells as described (65). Briefly, Sf9 cells were grown at 27 °C to a density of 2 to 3 × 10^6^ cells/ml in SF-900 II serum-free media (GIBCO) supplemented with 10% FBS and gentamycin (GIBCO). Baculovirus was added at a ratio of ten plaque forming units per cell and cells were harvested 46 to 48 h post infection by centrifugation at 4 °C (15 min, 1900 RPM) and washed once with ice cold PBS. All subsequent steps were done at 4 °C or on ice. The cell pellet was resuspended in lysis buffer (20 mM Hepes, pH 7.2, 250 mM NaCl, 0.02% Triton X-100, 5 mM EDTA, 1 mM DTT, 1 mM PMSF, 3 mM benzamidine, and 10.5 μM leupeptin), homogenized using a Brinkman Polytron, and centrifuged at high speed to clarify the lysate. The supernatant was diluted 4-fold in buffer A (20 mM Hepes, pH 7.2, 2 mM EDTA, 0.02% Triton X-100, and 1 mM DTT), centrifuged (20 min at 15,000 rpm), loaded onto an SP Sepharose cation exchange column, and eluted with a 50 to 500 mM NaCl gradient in buffer A. The GRK6-containing fractions were pooled, diluted in buffer A, loaded onto a heparin Sepharose 6 FF affinity column, and eluted with a 50 to 500 mM linear NaCl gradient in buffer A. GRK6-containing fractions were pooled, diluted with buffer B (20 mM Hepes, pH 7.2, 1 mM DTT), and injected onto a 1 ml Mono S cation-exchange FPLC column. Proteins were eluted with a 50 to 600 mM NaCl linear gradient in buffer B, and fractions containing GRK6 were combined, diluted to 200 mM NaCl with buffer B, concentrated in a 30 kDa cutoff filter (Millipore Corp) to 2 mg/ml, aliquoted, and stored at −80 °C. The purified protein was used for *in vitro* phosphorylation assays.

### *In vitro* kinetic analysis

The phosphorylation efficiency of WT GRK6 and GRK6-P384S was determined by incubating purified GRK6 (30 nM) with bovine rod outer segment membranes containing rhodopsin (0.5–10.5 μM) or with porcine tubulin (0.05–8 μM, Cytoskeleton Inc) in 100 μM ATP with [γ-^32^P]ATP (∼2000 cpm/pmol), 10 mM Tris–HCl, pH 7.4, 1 mM EDTA, 5 mM MgCl_2_, 25 mM NaCl, and 0.0004% Triton X-100 for 5 min at 30 °C. Reactions were stopped with SDS sample buffer and samples were separated by SDS-PAGE. Gels were stained with Coomassie blue, dried, audioradiographed, and substrate bands were excised and counted using a scintillation counter (Tri-Carb 4910 TR, PerkinElmer). Kinetic values were determined by fitting the data to Michaelis–Menten kinetics using GraphPad Prism (GraphPad Software Inc) from at least three independent experiments. Kinetic values for ATP were determined as aforementioned by incubation with ATP (12.5–200 μM) and tubulin (2 μM) for 3 min at 30 °C.

### GRK localization in HeLa cells

Coverslips were sterilized in 70% ethanol, placed into each well of a 6-well dish, and rinsed twice with sterile Dulbecco’s PBS (DPBS) without magnesium and calcium (Corning). HeLa cells were plated onto the coverslips to be 60% to 70% confluent at the time of transfection. Cells were transfected with 1 μg of DNA using Lipofectamine 2000 per the manufacturer’s protocol (Thermo Fisher), the media was replaced with complete media the next day, and cells grown for an additional 24 h. The coverslips were then washed with DPBS containing calcium and magnesium (Corning) and fixed for 20 min in 3.7% formaldehyde in DPBS containing magnesium and calcium. The fixed cells were then washed three times with DPBS containing magnesium and calcium and subsequently permeabilized for 20 min with 2 ml/well of blocking buffer (50 mM Tris–HCl, pH 7.5, 150 mM NaCl, 1% Triton X-100, and 2.5% nonfat dry milk). After permeabilization, the blocking buffer was aspirated and 50 μl of primary antibody (GRK4-6, Millipore, 1:200 in blocking buffer) was added to each coverslip for 1 h and the coverslips were then washed five times with blocking buffer, allowing each wash to sit for 5 min. Fifty microliters of secondary antibody (Alexa fluor 488–conjugated goat antimouse, Invitrogen, 1:500 in blocking buffer) was added for 30 min and the cells were washed five times for 5 min each in DPBS containing magnesium and calcium. The coverslips were then removed, rinsed in milli-q water, and the edges blotted dry. Each coverslip then received 15 μl of Prolong Gold Antifade Mount with 4′,6-diamidino-2-phenylindole (Invitrogen) and placed cell side down on a fresh slide. The slides were left at room temperature (RT) overnight in the dark and imaged within a week using a Nikon A1R Laser Scanning Confocal microscope.

### GRK6 localization in MIN6 cells

nt-shRNA MIN6 cells were plated at 1 × 10^6^ cells/well in 6-well plates and grown overnight. The following day, the cells were infected with negative control, WT human GRK6 or GRK6-P384S lentiviruses as per the manufacturer’s protocol (Genecopoeia). In brief, polybrene was diluted in MIN6 complete media to a final concentration of 8 μg/ml. The lentiviral constructs were then added to 1 ml aliquots of the polybrene mixture at volumes corresponding to 2 multiplicity of infection (MOI) to generate infection mixes. The media was aspirated from the cells, and 1 ml of polybrene media with no lentivirus was added per well. The infection mixes (1 ml) were then added dropwise to their corresponding wells and incubated at RT for 30 min. The day following infection, each original infection well was washed, trypsinized, and split into three new wells containing coverslips sterilized with 70% ethanol and washed with DPBS as described previously. The cells were allowed to grow for an additional 2 days for a total infection time of 72 h. The coverslips were then fixed and stained as described previously for HeLa cells. In these experiments, the anti-GRK6 rabbit mAb was used as the primary antibody (1:250 in blocking buffer). The Alexa fluor 488–conjugated goat anti-rabbit was used as the secondary antibody (1:500 in blocking buffer).

### Detection of cellular proteins by Western blotting

For intracellular protein level analysis, cells were washed in ice-cold DPBS with no cations (Corning) and lysed in lysis buffer (20 mM Hepes, pH 7.2, 250 mM NaCl, 0.02% Triton X-100, 5 mM EDTA, 1 mM DTT, 1 mM PMSF, 3 mM benzamidine, and 10.5 μM leupeptin) for 5 min. The cells were then scraped, snap frozen in liquid nitrogen, and the lysates stored at −20 °C. Lysates were thawed on ice, incubated for 30 min at 4 °C on a rocker, clarified by centrifugation (4 °C, 15,000 rpm), and diluted in lysis buffer to make 0.5 μg/μl protein samples as determined by Bradford assay. The level of proinsulin and insulin was determined as previously described with some modifications (66). Briefly, proteins were separated by SDS-PAGE on a 16.5% Tris-tricine gel (Bio-Rad) using a cathode buffer containing 0.1 M Tris–HCl, pH 8.8, 0.1 M Tricine, and 0.1% SDS and anode buffer containing 0.1 M Tris-HCl, pH 8.8. Gels were then transferred to 0.45 μM Immobilon-P polyvinylidene difluoride membranes (Millipore) for 45 min at 70 V. The membranes were preblocked in insulin blocking buffer (1% nonfat dry milk, 0.1% bovine serum albumin [BSA] in Tris-buffered saline with Tween-20 [TBS/T]) for 5 min, washed for 3 min with PBS with Tween-20 (PBS/T), and fixed in 0.2% glutaraldehyde (Sigma–Aldrich) in PBS/T for 15 min. The membranes were washed three times with PBS/T, microwaved for 10 min in citrate retrieval buffer (10 mM citric acid, pH 6, 1 mM EDTA, 0.05% Tween 20), cooled to RT, and then quenched with 200 mM glycine in PBS/T. The membranes were blocked for 30 min in insulin blocking buffer and incubated overnight with primary antibodies against insulin in 3% BSA, TBS/T. After three washing steps, membranes were incubated for 1 h in insulin blocking buffer containing the appropriate secondary antibodies, washed three times, incubated in SuperSignal West Pico chemiluminescent substrate (Protein Biology, Thermo Fisher Scientific), and then analyzed by exposure to film or by digital imaging using an Amersham Bioimager. Purified bovine insulin (Sigma–Aldrich) was used as a control to measure intracellular insulin levels with the insulin primary antibody. All quantitation was done using ImageJ (https://imagej.nih.gov/ij/download.html) or the imaging software on the Amersham Bioimager. Detection of other proteins used traditional immunoblotting techniques including 5% nonfat dry milk in TBS/T for blocking buffer. All primary antibodies were made in 3% BSA, TBS/T. The following primary antibodies were used: anti-insulin mouse monoclonal (Cell Signaling Technologies (CST), #8138S, 1:3000), anti-insulin rabbit monoclonal (CST, #3014S, 1:3000), anti-GRK6 rabbit monoclonal (CST, #5878, 1:3000), anti-PC1/3 rabbit polyclonal (CST, #11914, 1:3000), anti-PC2 rabbit monoclonal (CST, #14013, 1:3000), anti-CPE rabbit polyclonal (Abcam, #Ab11044, 1:5000), antitubulin mouse monoclonal (Millipore Sigma–Aldrich, #T5168, 1:10,000), and anti-GRK4-6 mouse monoclonal (Millipore Sigma–Aldrich, #05-466, 1:3000). The following secondary antibodies were used: goat anti-rabbit IgG antibody, peroxidase (Vector Laboratories, #P1-1000-1, 1:3000), and horse antimouse IgG, peroxidase (Vector Laboratories, #P1-2000-1, 1:3000). Total protein loading was determined by ponceau staining (Sigma Life Science, #P7170) following transfer and/or tubulin detection as described previously.

### Generation of GRK6 knockdown stable cell lines

MIN6 cells were infected with non-mammalian shRNA control transduction particles (Mission Sigma, #SHC002V, nt-shRNA) or shRNA transduction particles targeting five different portions of the *GRK6* gene. The following mission lentiviral transduction particles were used: TRCN0000022849 (shRNA-1), TRCN0000022851 (shRNA-2), TRCN0000022852 (shRNA-3), TRCN0000361581 (shRNA-4), and TRCN0000361508 (shRNA-5). Lentiviral transduction of MIN6 cells was carried out following the manufacturer’s protocol (Mission RNAi, Sigma–Aldrich). In brief, 1.6 × 10^4^ cells/well were plated into a 96-well plate and grown overnight. The following day, media was removed and each well received 110 μl of normal complete media containing 8 μg/ml hexadimethrine bromide (also known as polybrene, Sigma–Aldrich). Each construct was designated 6 wells of a 96-well plate: 1 to receive no lentiviral transduction particles, 1 to receive hexadimethrine bromide only, and the other four to receive varying amounts of lentiviral transduction particles (2, 5, 10, 15 μl) in complete medium containing hexadimethrine bromide. The following day, the media was removed and replaced with 120 μl of fresh normal complete media with no hexadimethrine bromide. After 48 h of transduction, the media was replaced with complete media containing 2.5 μg/ml puromycin (knockdown complete media) to begin selection of stable clones. Every 3 to 4 days, fresh knockdown complete media was added to the cells until resistant colonies were identified (∼12 days). Wells were chosen where only a few clones remained. For each construct, these clones were pooled together and scaled up in larger cell culture dishes until they could be frozen down and assayed by Western blot to detect the level of GRK6 knockdown. shRNA-2 and shRNA-5 stable cell lines were used for most experiments as they had the highest level of GRK6 knockdown.

### GSIS assays

MIN6 cells were plated at 1.5 × 10^6^ cells/well in 6-well plates for glucose dose responses with 3 to 6 concentrations (Figs. 1, 2, *C* and *D*, 3, *B*–*E*) or 1 × 10^6^ cells/well in 12-well plates for glucose dose responses with eight concentrations (Figs. 2, *A* and *B*, 3). The following day, the cells were washed with Krebs-Ringer bicarbonate/Hepes (KRBH) containing 0.008 to 0.01 mM glucose and pretreated in the same buffer for 30 min. The cells were washed again with KRBH containing 0.008 to 0.01 mM glucose and then placed in KRBH buffer containing various glucose concentrations up to 18 mM with or without 1 μM compound 19 for 1 h. In the experiments using GRK6 knockdown cells, shRNA-2 and shRNA-5 cells were not treated with any inhibitors. The amount of proinsulin and insulin secreted was determined by measuring the proinsulin or insulin levels in the media by ELISA (Caymen Chem in Fig. 1, ALPCO for everything else). Intracellular protein detection was done by Western blotting as described previously for proinsulin, insulin, PC1, PC2, CPE, tubulin, and the GRKs.

### GRK6 rescue in knockdown MIN6 cells

Both nt-shRNA and shRNA-5 cells were plated at 1 × 10^6^ cells/well in 6-well plates. The following day, the cells were infected with either negative control or GRK6 lentiviruses encoding human GRK6 as per the manufacturers protocol (Genecopoeia). In brief, polybrene (Sigma–Aldrich) was diluted in knockdown complete media to a final concentration of 8 μg/ml. The lentiviral constructs were then added to 1 ml aliquots of the polybrene mixture at volumes corresponding to the desired MOI to generate infection mixes. The media was aspirated and 1 ml of polybrene media with no lentivirus was added per well. The infection mixes (1 ml) were then added dropwise to their corresponding wells, incubated at RT for 30 min, and then placed back in the incubator. For experiments with complete media treatment, cells were infected at an MOI of 1.7 and the cell media was replaced the day following infection. Two days post infection, each original well was washed, trypsinized, and split into two new wells of a 6-well dish to provide a well for both treatments, 0.01 mM glucose in KRBH (−) *versus* complete media (+). The cells were grown for another day before treatment for a total of 72 h of infection. Insulin secretion and intracellular protein determination were done as described previously but with the (+) treatment being that of complete media (25 mM glucose, 15% FBS) instead of glucose in KRBH. For GSIS experiments lasting 2 h, cells were infected at an MOI of 2, and each infected well was split the day following infection into three new wells.

### Extended glucose treatment in GRK6 overexpressing MIN6 cells

MIN6 cells were plated at 1 × 10^6^ cells/well in a 6-well plate and grown overnight. The following day, the cells were infected with negative control, WT GRK6, GRK6-P384S, or GRK6-K215R lentiviruses encoding human GRK6 as per the manufacturer’s protocol (Genecopoeia). In brief, polybrene was diluted in MIN6 complete media to a final concentration of 8 μg/ml. The lentiviral constructs were then added to 1 ml aliquots of the polybrene mixture at volumes corresponding to the desired MOI to generate infection mixes. The media was aspirated from the cells and 1 ml of polybrene media with no lentivirus was added per well. The infection mixes (1 ml) were then added dropwise to their corresponding wells and incubated at RT for 30 min. The day following infection, each original infection well was washed, trypsinized, and split into three new wells per each glucose treatment. These were allowed to grow for an additional 2 days for a total infection time of 72 h. GSIS and intracellular protein determination was then conducted as described previously with the exception that the time of treatment was 4 h rather than 1 h. Secreted insulin levels were not measured in these experiments.

### Antibody validation

The anti-insulin mouse monoclonal and anti-insulin rabbit monoclonal used in Figs. 2, *C* and *D*, 3, *B* and *C*, and 9*A* were previously validated (66, 67). The anti-GRK4-6 mouse monoclonal used in Figs. 3*A* and 7 and anti-GRK6 rabbit monoclonal used in Figs. 3*A*, 5, *A* and C, 8*A*, and 9*A* were validated by GRK6 knockdown as shown in Fig. 3*A*. The anti-PC1/3 rabbit polyclonal, anti-PC2 rabbit monoclonal, and anti-CPE rabbit polyclonal were previously validated (68, 69, 70).

### Statistical analysis

All statistical analyses were done using GraphPad prism version 9.3.1. All data are shown as the mean ± SD from at least three independent experiments except for Table 1, which is presented as the mean ± SEM. Each figure legend states the specific statistical test that was done for each experiment and the number of replicates. ∗*p* < 0.05; ∗∗*p* < 0.01; ∗∗∗*p* < 0.001 in all analyses.

## Data availability

All study data are included in the article.

## Conflict of interest

The authors declare that they have no conflicts of interest with the contents of this article.
